# Bibliometric and Visualization Analysis of Research on Exosomes as Drug Delivery Systems (2008-2023)

**DOI:** 10.2174/0115672018358562250213113042

**Published:** 2025-02-20

**Authors:** Wei Xiang, Qisong Shang, Zhoujun Zhu, Yuanyuan Wu, Xinghua Song

**Affiliations:** 1 Department of Spine Surgery, The Sixth Affiliated Hospital of Xinjiang Medical University, Urumqi, Xinjiang, China;; 2 Department of Joint Surgery, The Sixth Affiliated Hospital of Xinjiang Medical University, Urumqi, Xinjiang, China

**Keywords:** Bibliometrics, CiteSpace, VOSviewer, exosome, drug delivery, research hotspots, research trends

## Abstract

**Introduction:**

Exosomes are unique bio-nanomaterials possessing significant value and potential for drug delivery systems. However, to date, no bibliometric studies in this field have been reported. Our aim is to explore the research hotspots and trends of exosome drug-carrying systems across various medical fields through bibliometric analyses.

**Methods:**

Articles and reviews related to “exosome” and “drug delivery” are retrieved from the Web of Science Core Collection. VOSviewer, CiteSpace, Scimago Graphica, and Origin 2021 are employed for bibliometric analyses.

**Results:**

A total of 771 articles from 60 countries, such as China and the United States, are included. The number of papers concerning exosomal drug delivery systems has been increasing yearly. The main research institutions are the Chinese Academy of Sciences, Shanghai Jiao Tong University, Huazhong University of Science and Technology, Fudan University, and Sichuan University. The Journal of Controlled Release is the most prevalent and frequently cited journal in this field. These papers are authored by 247 individuals, with Ando, Hidenori having the highest number of publications and Alvarez-Erviti L receiving the most citations. “Extracellular vesicles”, “drug delivery”, “*in vitro*”, “nanoparticles”, “cells”, “delivery”, and “mesenchymal stem cells” are the principal keywords for this hotspot.

**Conclusion:**

This pioneering bibliometric study offers a comprehensive overview of the research trends and advancements in exosomal drug delivery systems in medicine over the past fifteen years.

## 1 INTRODUCTION

Exosomes, with a diameter of approximately 30-150 nm, are nanospherical lipid bilayer vesicles of biological origin that are secreted by cells and band at a density ranging from 1.13 to 1.19 g/mL in a sucrose density gradient solution [1-4]. In 1981, Trams *et al*. [5] coined the term 'exosomes' to refer to vesicles originating from the plasma membrane as exosomes and initially introduced the concept of exosomes. They are regarded as membrane vesicles possessing 5'-nucleotidase activity, which might have physiological functions stemming from cytosolic behaviors in a diverse array of cell lines [6-8]. The exosomes as currently defined were initially identified in 1983 for the first time in sheep reticulocytes. Their biogenesis begins with endocytosis by cells to form early endosomes, which then transform into multivesicular bodies. After the multivesicular bodies fuse with the cell membrane, exosomes are released [9]. Exosomes have been found to contain nucleic acids, proteins, lipids, cytokines, transcription factor receptors, and other bioactive substances, this would be the basis on which exosomes could be used as drug delivery systems [10-13].

Exosomes are capable of playing a role in both physiological and pathological processes, functioning as mediators for intercellular communication as well as material exchange [14, 15]. Exosomes can participate in intercellular communication and transmit bioactive molecules to regulate the physiological activities of recipient cells. In particular, they play an important role in the communication of cancer cells [16-18]. For example, in immune regulation, they mediate the signal transduction among immune cells [19]. In the tumor microenvironment, they affect tumor progression by transporting oncogenic or tumor-suppressive substances [1, 20, 21]. Moreover, they also play a role in various pathological processes, such as neurodegenerative diseases and cardiovascular diseases. Based on the above functions, exosomes have become effective therapeutic targets and drug delivery platforms [2, 22]. Simultaneously, exosomes are capable of delivering a diverse range of bioactive substances and components that are readily inactivated or degraded through multiple pathways and sites, and safely transferring them to target cells, thereby participating in tissue repair, tumour diagnosis and treatment, as well as immune regulation [1, 23]. Consequently, exosome drug-carrying systems have been increasingly developed, especially in the field of oncology. In 2013, Jang, SC *et al*. developed bionic exosome-mimicking nanovesicles, which could deliver chemotherapeutic drugs to tumour tissues following systemic administration [24]. The adriamycin-loaded nanovesicles exhibited comparable *in vivo* antitumour activity to doxorubicin-loaded exosomes. In recent years, based on exosome drug-carrying systems, engineered exosomes can not only achieve drug loading but also enable targeted transport, thus enhancing therapeutic efficacy [25-27].

Bibliometrics represents an analytical approach used to visualize literature, enabling qualitative and quantitative analyses of scientific research outcomes within a specific field over a defined time period [28-30]. We can examine the detailed information of authors, keywords, journals, countries, institutions, and references [31-33], utilize the bibliometric tools CiteSpace 6.2.7 and VOSviewer to visualize the co-occurrence and emergence of each indicator of the analysis results, and also generate dual-map overlays, enabling readers to intuitively understand the results. These tools have been widely employed in scientific research. Wang B *et al*. carried out a visualization and analysis of global exosome research [28]; however, the study did not present detailed research progress regarding exosome drug-carrying systems. The relevant literature outputs in recent years have suggested that exosome drug delivery systems hold promising research prospects [34-36], indicating a knowledge gap. Therefore, the objective of this study was to conduct a visual bibliometric analysis of the scientific output of exosomes in medicine over the past fifteen years (2008 - 2023), to identify the major contributors and the current research status, and to explore the research trends and future prospects of this field.

## 2 METHODS

### 2.1 Search Strategy

Web of Science is a widely used and authoritative academic literature database covering high-quality journals and conference papers in numerous fields and other resources. Its important position and wide recognition in academic research are some of the main reasons for selecting it. In the early stage of the research, we integrated the resources of multiple databases. However, the considerable variations among different databases in areas, such as literature classification, indexing methods, and data formats, introduced significant complexity and uncertainty to the screening, sorting, and analysis of data, which seriously affected the efficiency and feasibility of the research. After careful consideration, we finally decided to concentrate on delving deeply into the data in Web of Science to ensure that systematic and in-depth research and analysis could be carried out under relatively unified data standards, thus maximizing the consistency of research methods and the reliability of results.

**Fig. (1):**
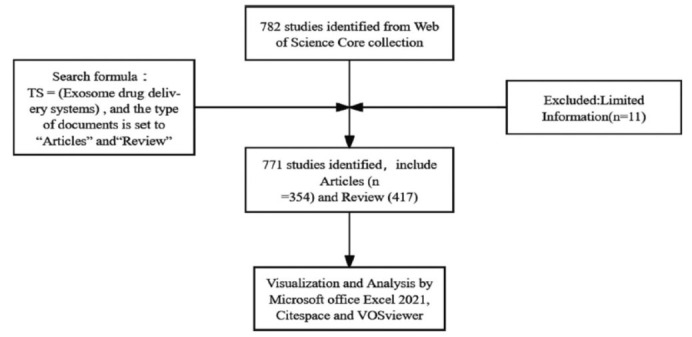
Flowchart of literature retrieval.

A comprehensive literature search was carried out on the Web of Science Core Collection (https://www.webofscience.com/wos/woscc) during the period spanning from 2008 to 2023. The search formula employed was ts = (“exosome” and “drug delivery”), and the document types were limited to “Articles” and “Review” (Fig. **1**). Exosome is a type of extracellular vesicle. Other types include microvesicles (MVs) or microparticles and apoptotic bodies. This study investigates the trends and hotspots of exosomes as a drug delivery system. Moreover, the exosome is also the extracellular vesicle that is most frequently used to construct drug delivery systems. Therefore, when conducting literature retrieval, the papers with microvesicles, microparticles, and apoptotic bodies as the main topics were excluded.

### 2.2 Data Analysis

VOSviewer is a software tool designed for visualizing and analyzing citation networks. It enables the exploration and mapping of knowledge domains by generating network maps and conducting diverse types of analyses on literature data, including network visualization, density visualization, keyword analysis, cluster analysis, and citation metrics analysis [37, 38]. We utilized this software to visualize and analyze countries and institutions, journals and co-cited journals, authors and co-cited authors, as well as keyword co-occurrence. For co-occurrence analysis, the minimum count was set at 5, and only countries, institutions, and journals that appeared at least 5 times in the retrieved literature were considered. This approach was used to filter out low-frequency research objects and minimize excessive interference. The node scaling was set to “linear”, and the node size was adjusted according to the node's indicators to reflect its importance.

CiteSpace represents an extension and enhancement of VOSviewer, with its emphasis placed on the exploration of relational networks and the evolutionary examination of knowledge domains. It visualizes the citation linkages among documents and uncovers the structural and evolutionary patterns within research domains by constructing citation network graphs [39, 40]. The software was employed to analyze the emergence of co-cited authors, co-cited journals, co-cited documents, and keywords, as well as to generate keyword time-zone diagrams and double-stacked charts. Before constructing the co-occurrence network, the length of each time slice was set to 2, indicating that each time slice represents a period of two years so as to observe the evolution of research within different time periods. The number of selected nodes within each time slice was set, with Top *N =* 50, indicating that the 50 most highly cited works of literature, authors, or journals within each time slice were selected to ensure that the analyzed nodes have relatively high influence. The display mode of node labels was set to “By Degree” (displayed based on node degree), and only the labels of important nodes were shown to avoid confusion caused by an excessive number of labels. The Kleinberg algorithm was used to detect the burstiness of keywords, literature, or authors in order to identify elements that suddenly emerge or attract attention in the research field. Burstiness can help identify emerging research hotspots or suddenly rising research forces. The hierarchical clustering algorithm was used for cluster analysis, which could provide abundant hierarchical information and handle small-scale data structures more meticulously.

Scimago Graphica is used to draw geographical maps. It can combine data with maps based on the distribution of data in geographical areas and intuitively display data differences in different regions, such as the scientific research cooperation networks and the distribution of scientific research achievements in different countries or regions.

## 3 RESULTS

### 3.1 Analysis of the Number of Publications and Citations in the Literature

**Fig. (2):**
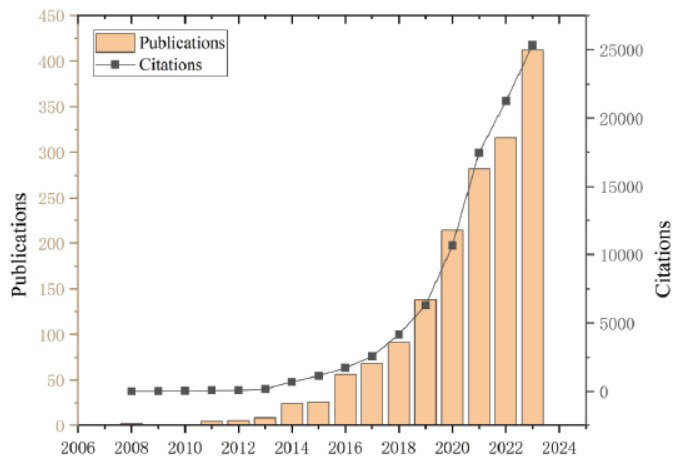
Trends in the number of publications and citations over the past fifteen years.

In accordance with our search criteria, a total of 771 studies regarding exosomes in drug delivery systems were published over the past 15 years, among which 354 were “articles” and 417 were “reviews”. Both the number of publications and citations exhibited an upward trajectory on an annual basis, and the number of publications in each period mirrored the contemporary development of research within this field (Fig. **2**). The time frame could be broadly segmented into three phases: the first phase from 2008 to 2011, the second phase from 2012 to 2016, and the third phase from 2017 to 2023. During the first phase, the number of studies remained relatively stable as the exosome drug delivery system was only beginning to uncover its potential. The second phase witnessed a moderate growth trend, and the third phase was characterized by a steady and continuous increase in the number of publications and citations year by year, reaching its zenith precisely in 2023. Currently, researchers are actively probing into the potential of exosomes as drug delivery systems.

### 3.2 Country and Institutional Analysis of Publications

These publications stemmed from diverse institutions across the globe, and the top ten countries and institutions are presented in Table **1**. The country with the largest number of publications was China (*n =* 343, accounting for 44.49%), followed by the United States (*n =* 155, 20.10%), Japan (*n =* 61, 7.91%), Iran (*n =* 59, 7.65%), and South Korea (*n =* 53, 6.88%). Both China and the United States boasted the highest number of publications, with each having over 100.

The research institution that had the greatest number of publications was the Chinese Academy of Sciences (*n =* 22, 2.853%), followed by Shanghai Jiao Tong University (*n =* 19, 2.464%), Huazhong University of Science and Technology (*n =* 18, 2.335%), Fudan University (*n =* 16, 2.075%), and Sichuan University (*n =* 16, 2.075%). These five institutions accounted for 70% of those within the top ten institutions.

**Fig. (3):**
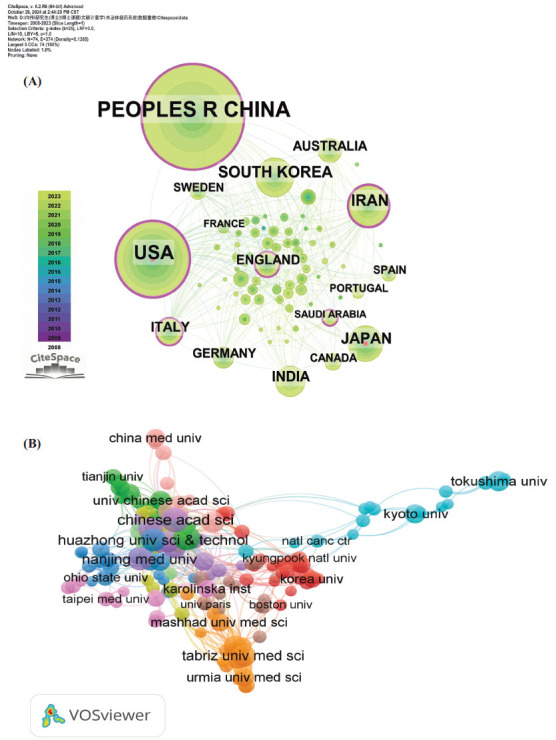
Co-occurrence analysis of the countries (**A**) and institutions (**B**) in the literature on exosomes as drug delivery systems.

The countries and institutions with the number of published articles greater than or equal to 3 were identified (Figs. **3A**-**B**), and close cooperation was found among different countries and institutions.

### 3.3 Analysis of Publication Journals and Cited Journals

**Fig. (4):**
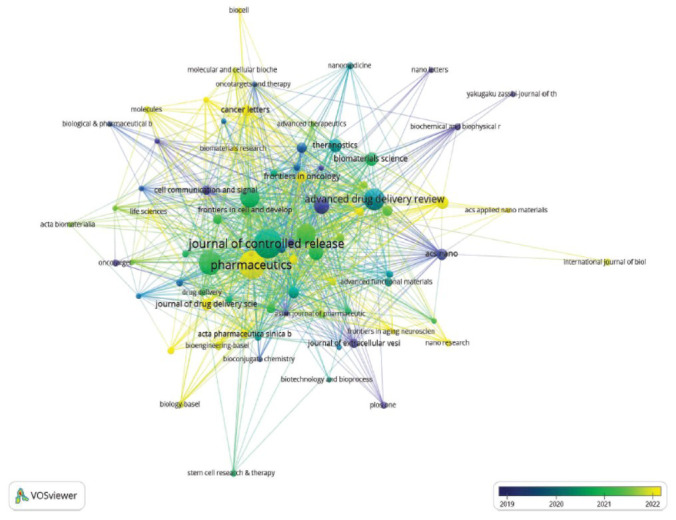
Co-occurrence analysis of the journals in which the literature on exosomes as drug delivery systems was published.

Based on the network co-occurrence graph of the publishing journals, it was observed that the Journal of Controlled Release was not only the journal with the highest number of articles (Fig. **4**) but also the most frequently cited journal (*n =* 589). It was followed by Biomaterials (*n =* 545) and Scientific Reports (*n =* 503). They were the only three journals that received more than 500 citations (Table **2**). The journal with the highest impact factor was ACS Nano (IF = 17.0997), trailed by Nature Communications (IF = 16.6009) and Advanced Drug Delivery Reviews (IF = 16.1004). Moreover, the Journal of Controlled Release was also the journal that had the closest inter-citation relationship with other journals.

**Fig. (5):**
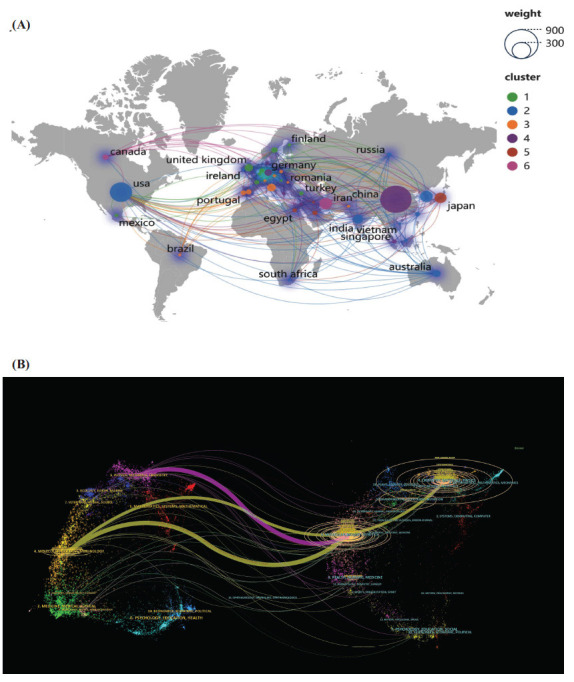
Geographical map of the journals that published articles on exosomes as drug delivery systems (**A**) and the dual-map of co-cited journals (**B**).

The geographical map and dual-map overlay of journals displayed the cross-citation relationship between citing journals and cited journals (Figs. **5A** and **B**), with the citing journals positioned on the left and the cited journals on the right. As depicted, the yellow path represents the primary citation path, indicating that research published in chemistry/materials/physics/molecular/biology/genetics journals is predominantly cited by literature in molecular/biology/immunology journals.

### 3.4 Analysis of Cited References for Publications

**Fig. (6):**
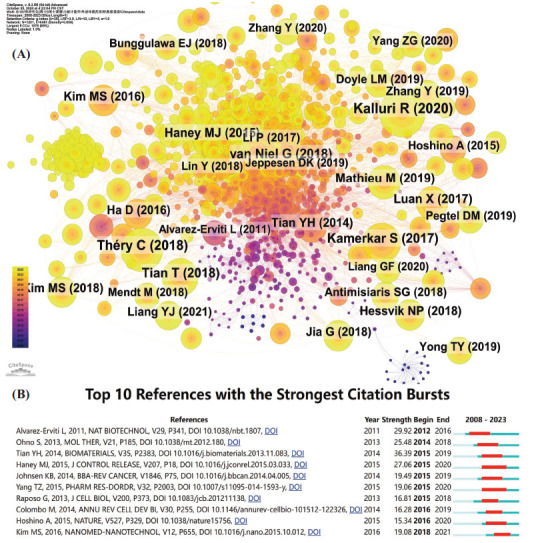
Co-occurrence analysis of the cited references in the research on exosomes as drug delivery systems (**A**) and the top 10 references with the strongest citation bursts (**B**).

The paper that received the highest number of citations was authored by Kalluri R. and published in 2020 (*n =* 148, IF = 41.845) (Table **2**). It was followed by the paper published by Théry C in 2018 (*n =* 122, IF = 14.976). An article published in Nature (authored by Kamerkar S in 2017) was also cited over 100 times. Moreover, the reference network co-occurrence graph revealed that the citation status of each reference was relatively balanced, and there was no particularly prominent node (Figs. **6A** and **B**).

We utilized Citespace to analyze the emergence of references and listed the 10 papers with the highest burst strength. Among them, the first one was published by Tian Yh in Biomaterials in 2014 (Strength = 36.39), reflecting the high popularity of this paper during the period from 2015 to 2019. Overall, the intensity of these 10 literature bursts ranged from 19.08 to 29.92, with a persistence time spanning 3 to 5 years.

### 3.5 Analysis of Publication Authors and Cited Authors

Among the top ten authors ranked by the number of publications, two authors tied for the first place (*n =* 9), each having an identical number of publications (Table **3**). They were followed by six authors who each had four publications.

**Fig. (7):**
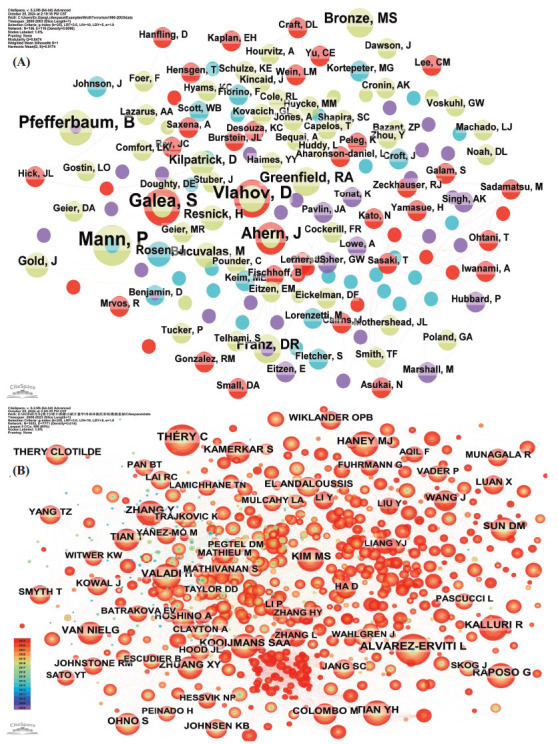
Co-occurrence analysis of the authors of published literature (**A**) and co-occurrence analysis of the cited authors (**B**) in the research on exosomes as drug delivery systems.

The close cooperation among the authors could also be observed in the network co-occurrence graph (Fig. **7A**). The most cited author was Alvarez-Erviti L (*n =* 302), who was the sole author with more than 300 citations (Fig. **7B**). This author was followed by Théry C (*n =* 280), Haney Mj (*n =* 208), Tian Yh (*n =* 204), and Kooijmans Saa (*n =* 202).

### 3.6 Publication Keyword Analysis

We selected 20 high-frequency keywords (Table **4**). Among them, the keyword with the highest frequency was “extracellular vesicles” (*n =* 366), followed by “drug delivery” (*n =* 264), “*in vitro*” (*n =* 149), “nanoparticles” (*n =* 132), “cells” (*n =* 108), “delivery” (*n =* 107), and “mesenchymal stem cells” (*n =* 106). The frequencies of the remaining keywords were less than 100.

**Fig. (8):**
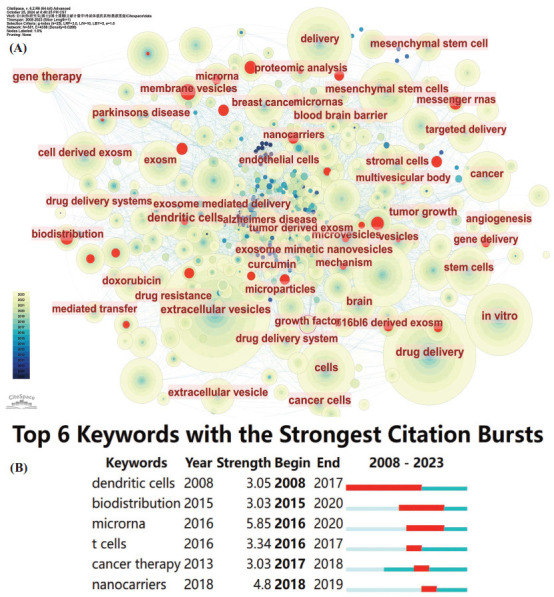
Co-occurrence analysis of keywords (**A**) and the top 6 keywords with the strongest citation bursts (**B**) in the research on exosomes as drug delivery systems.

We employed the software to analyze the network co-occurrence of the keywords. It can be observed that the nodes with the largest sizes are the top-ranked keywords in the table, and the connections between different keywords are dense, which indicates a high degree of correlation among them (Fig. **8A**). Subsequently, we utilized Citespace to conduct an emergence analysis on the keywords and filtered out six keywords with the highest burst strength (Fig. **8B**). These keywords were “dendritic cells” (Strength = 3.05), “biodistribution” (Strength = 3.03), “miRNA” (Strength = 5.85), “T cells” (Strength = 3.34), “cancer therapy” (Strength = 3.03), and “nanocarriers” (Strength = 4.8). The keyword with the greatest burst strength was “miRNA” (Strength = 5.85), followed by “nanocarriers” (Strength = 4.8), suggesting that it became the most popular keyword during the period from 2016 to 2020.

**Fig. (9):**
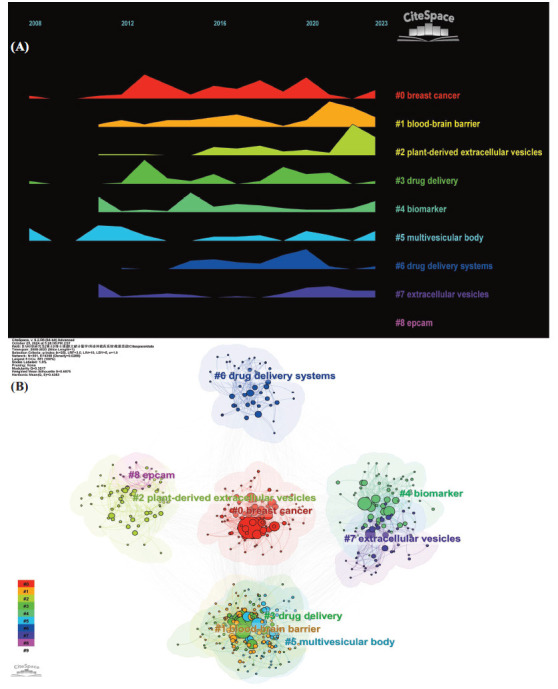
Map of keyword time zones (**A**) for the study of exosomes as drug delivery systems and cluster analysis (**B**).

We utilized Citespace to draw a keyword time zone graph (Fig. **9A**) to visualize the keywords along with the corresponding years in which they emerged. It is evident that both the number and types of keywords have been increasing over time, which indirectly implies that the research on exosome drug delivery systems has been advancing. Subsequently, the keywords were visualized through cluster analysis (Fig. **9B**). In this analysis, all keywords were grouped into 11 categories, and the visualization was plotted in relation to time. The largest nodes represent the first major category (“cells”), seemingly indicating the most intensive research on exosomal drug-carrying systems in this area. The time zone and clustering analyses of keywords assisted us in quickly identifying the current research hotspots and cutting-edge research directions, as well as predicting the research evolution trends.

## 4 DISCUSSION

Bibliometric analysis serves as a tool for quantitatively analyzing publications. It enables the assessment of the quality and impact of scientific research, facilitates the study of disciplinary trends and the structure of fields, and allows for the analysis of the impact of journals, authors, and institutions [41-43]. A global visualization of studies on exosomes from 2007 to 2016 was conducted in the existing literature [44]. Moreover, some authors have recently carried out a bibliometric study on exosomes in ischaemic stroke [45, 46]. It has also been analyzed in other systemic diseases, such as cardiovascular disease [47, 48], osteoporosis [49-51], pancreatic cancer [52, 53], and Parkinson's disease [54, 55]. However, up to now, no bibliometric analysis has been performed regarding the utilization of exosomes as a drug-carrying system based on the properties of their nanomaterials. The present study is the first to carry out a bibliometric analysis of exosomes as a drug-carrying material and perform visualization to analyze the quality and quantity of research.

In recent years, nanomaterials have emerged and witnessed significant progress. Particularly, biological nanomaterials like exosomes can be applied in diverse fields, such as cardiovascular diseases [56-59], tumours [60-63], diabetes [64-66], and osteoarthritis [67-69]. The study of employing them as a drug carrier system for precise drug delivery has also garnered substantial interest from researchers. We conducted a systematic literature search on the Web of Science (WOS) core database, which can offer precise data across all areas with strong recognition and uniform citation rates. Our results revealed that after a small peak in the research literature on exosome drug-carrying systems in 2016, there was a relative decline in 2017. Nevertheless, it has been in steady growth from 2017 to the present. This trend indicates that the development of exosome drug-carrying system research is currently in a stage of rapid progress and has attracted increased attention from the global medical community.

It was found that 87.03% of the total number of publications were published by five countries (China, the United States, Japan, Iran, and South Korea), while China and the United States accounted for 64.59%. This suggests that global research on exosome drug delivery systems is concentrated in large populated countries. The factors contributing to this uneven development are complex, with disease incidence, population size, and research status all playing significant roles in the differences in scientific output among countries. The citation frequency of a paper can mirror its quality. In 2020, a review by Raghu Kalluri published in Science received the highest number of citations [70]. This review elaborated in detail on the occurrence and origins of exosomes, as well as the current developmental status within various fields. Théry C, in collaboration with 384 other authors, updated the MISEV2014 guidelines to MISEV2018, which was published in the high-quality “Journal of Extracellular Vesicles” [71] and ranked second in terms of citations. This update laid a solid theoretical foundation for subsequent research on exosomes. Ranking third, also in a high-quality journal, Sushrut Kamerkar [71] utilized engineered exosomes carrying small interfering RNAs to target cancer cells for the treatment of pancreatic cancer, representing the highest-quality study to date regarding the application of exosome drug-carrying systems. Among the top 10 cited papers, only 3 were from China, and their citation frequencies were relatively low. Although China has the largest number of papers, the United States has consistently been at the forefront of this field, with the highest quality. This implies that the quality of Chinese literature requires improvement. However, the impact of Chinese research is more significant than that of other countries, which should not be overlooked.

Undoubtedly, the Journal of Controlled Release is the most recognized journal [72-75]. It not only features the highest number of articles but also boasts the highest number of citations. As a leading journal in the field of pharmacology, its quality is beyond doubt, and it encompasses numerous studies on exosomes as drug delivery systems [76-79].

Through the co-occurrence, emergence, time zone, and clustering analyses of keywords, we discovered that the keywords related to the research on exosome drug delivery systems continuously stem from various disciplines and fields and increase year by year. Particularly in the field of oncology, they are employed for the targeted treatment of cancer cells, such as those in breast cancer [80-83], lung cancer [84-87], and oral cancer [88], and are also utilized for targeting immune cells [89, 90]. Citespace grouped the keywords into 11 categories, and behind the rankings of “cells” and “biomimetic nanoparticles”, there is a category of “targeted delivery”. In recent years, the research on engineered exosomes has become more sophisticated, with exosomes being modified to possess specific functions through genetic engineering and chemical methods [25, 91-94]. It is anticipated that this field will become a research hotspot in the future when the research on exosome drug delivery systems is expected to achieve a qualitative leap.

In summary, the current hotspot within the field of exosome drug delivery systems lies in the loading and targeting of drugs in cancer research. This represents a highly promising and valuable direction for utilizing exosomes as a drug delivery system. Presently, significant breakthroughs have been achieved in loading various chemotherapeutic drugs (such as paclitaxel) into exosomes. However, numerous related fields still hold considerable research value. For instance, loading different miRNAs and differential proteins into exosomes for the purpose of cell or tissue targeting and regulation remains an area worthy of in-depth exploration [95, 96]. Sha Li *et al*. [97] used peptides to modify exosomes in order to further improve tumor targeting so as to target the overexpressed mesenchymal-epithelial transition factor in TNBC cells. The results reported that the engineered exosomes coated with nanoparticles performed significantly in improving the cellular uptake efficiency of doxorubicin and the anti-breast cancer cell efficacy. The exosome itself has many kinds of miRNAs and proteins. If we can improve the miRNA loading into the exosome targeting regulation, it will change the biological behavior of cells or tissues at the gene and protein level. The research conducted by Hongxing Hu *et al*. reviewed that small extracellular vesicles derived from human umbilical cord mesenchymal stem cells (HUC-MSCs-sEVs) can activate the PTEN/AKT signaling pathway by delivering miR-23a-3p, thus promoting calcium deposition and the formation of endothelial networks, and finally inducing osteogenic differentiation and angiogenesis [98]. Currently, there are fewer studies on this topic, and no progress has been made.

Hojun Choi *et al*. reviewed the transport of exosomes expressing ligands (such as apolipoprotein B targeting LDLR), mediated by receptor-mediated transcytosis (RMT), which has shown promising results and elucidated the underlying mechanisms of BBB crossing by surface-modified exosomes [99]. Due to the existence of the blood-brain barrier (BBB), it is very difficult to deliver therapeutic drugs to the central nervous system (CNS) [100-102]. However, through the targeted modification of nanoscale exosomes to enable them to carry drugs into the CNS, this approach will overcome the problem and provide new therapeutic directions for many CNS diseases [103, 104]. For example, inspired by brain-targeting exosomes, Ruoning Wang *et al*. designed a biomimetic blood-brain barrier (BBB) penetrating hybrid (pHybrid) nanovesicle through the membrane fusion between blood exosomes and liposomes modified with tLyp-1 peptide, which can be used for brain-targeted drug delivery [105].

In this study, all relevant works of literature from specific databases were selected, and relevant information from the literature, such as authors, countries, institutions, journals, keywords, citation frequencies, *etc*., was collected. The software was utilized to draw maps like co-occurrence networks and author cooperation networks, which intuitively demonstrated the structure of the research field to ensure the rationality of the research design and the scientific nature of the experimental methods. During the data processing stage, data cleaning steps were carried out to remove the literature information with duplicate records, incorrect records, or incomplete records. The data, such as citation frequencies, were standardized to eliminate the influence of factors like the citation habits of different journals, ensuring the accuracy of data analysis. Exosomes have unique advantages as a drug delivery system, and bibliometric research on them is currently limited. Our study provides some insights into the research directions and hotspots in this field. Moreover, three kinds of software were combined for analysis, which, compared with using a single software, ensured the accuracy and usability of data analysis.

## CONCLUSION

As exosomes are studied in greater depth, they possess significant research value and hold promising application prospects in drug-carrying systems. In terms of both the quantity and quality of research output, China and the United States have made the most substantial contributions. Nevertheless, continuous efforts are required to enhance cooperation and communication among countries and institutions. For subsequent research directions, it is necessary to conduct an in-depth exploration, focusing on aspects like drug loading while also expanding it to other promising areas, such as miRNA and protein loading.

## Figures and Tables

**Table 1:** Top 10 countries and institutions for research on exosomal drug delivery systems.

**Rank**	**Country**	**Counts**	**Affiliations**	**Counts**
1	PEOPLES R CHINA	343(44.49%)	CHINESE ACADEMY OF SCIENCES	22(2.853%)
2	USA	155(20.10%)	SHANGHAI JIAO TONG UNIVERSITY	19(2.464%)
3	JAPAN	61(7.91%)	HUAZHONG UNIVERSITY OF SCIENCE TECHNOLOGY	18(2.335%)
4	IRAN	59(7.65%)	FUDAN UNIVERSITY	16(2.075%)
5	SOUTH KOREA	53(6.88%)	SICHUAN UNIVERSITY	16(2.075%)
6	ITALY	32(4.15%)	TOKUSHIMA UNIVERSITY	16(2.075%)
7	INDIA	29(3.76%)	UNIVERSITY OF CALIFORNIA SYSTEM	15(1.946%)
8	AUSTRALIA	22(2.85%)	NANJING MEDICAL UNIVERSITY	14(1.816%)
9	ENGLAND	21(2.72%)	TABRIZ UNIVERSITY OF MEDICAL SCIENCE	14((1.816%)
10	GERMANY	16(2.08%)	ZHEJIANG UNIVERSITY	13(1.686%0

**Table 2:** Top 10 cited journals and literature on exosomal drug delivery system research.

**Rank**	**Cocited Journal**	**Counts**	**IF**	**Cocited reference**	**Citations**	**IF**
1	J CONTROL RELEASE	589	10.7997	Kalluri R, 2020, SCIENCE, V367, P640, DOI 10.1126/science.aau6977	148	41.845
2	BIOMATERIALS	545	13.9993	Théry C, 2018, J EXTRACELL VESICLES, V7, P0, DOI 10.1080/20013078.2018.1535750	122	14.976
3	SCI REP-UK	503	4.6000	Kamerkar S, 2017, NATURE, V546, P498, DOI 10.1038/nature22341	109	42.778
4	MOL THER	483	12.4002	van Niel G, 2018, NAT REV MOL CELL BIO, V19, P213, DOI 10.1038/nrm.2017.125	106	55.47
5	J EXTRACELL VESICLES	481	15.9992	Tian T, 2018, BIOMATERIALS, V150, P137, DOI 10.1016/j.biomaterials.2017.10.012	100	10.317
6	PLOS ONE	461	3.7001	Luan X, 2017, ACTA PHARMACOL SIN, V38, P754, DOI 10.1038/aps.2017.12	85	5.064
7	NAT COMMUN	456	16.6009	Tian YH, 2014, BIOMATERIALS, V35, P2383, DOI 10.1016/j.biomaterials.2013.11.083	78	10.317
8	ACS NANO	441	17.0997	Mathieu M, 2019, NAT CELL BIOL, V21, P9, DOI 10.1038/s41556-018-0250-9	78	20.042
9	ADV DRUG DELIVER REV	433	16.1004	Kim MS, 2018, NANOMED-NANOTECHNOL, V14, P195, DOI 10.1016/j.nano.2017.09.011	77	5.182
10	NATURE	428	64.8001	Haney MJ, 2015, J CONTROL RELEASE, V207, P18, DOI 10.1016/j.jconrel.2015.03.033	75	7.727

**Table 3:** Top 10 authors and highly cited authors in terms of publications in exosomal drug delivery system research.

**Rank**	**Authors**	**Counts**	**Rank**	**Cocited Authors**	**Counts**
1	Ando, Hidenori	9	1	ALVAREZ-ERVITI L	302
2	Ishida, Tatsuhiro	9	2	THÉRY C	280
3	Liang, Yujie	4	3	HANEY MJ	208
4	Shimizu, Taro	4	4	TIAN YH	204
5	Ishima, Yu	4	5	KOOIJMANS SAA	202
6	Batrakova, Elena V	4	6	KIM MS	193
7	Xia, Jiang	4	7	SUN DM	184
8	Ochiya, Takahiro	4	8	KALLURI R	178
9	Takakura, Yoshinobu	3	9	RAPOSO G	176
10	Akiyoshi, Kazunari	3	10	VALADI H	174

**Table 4:** Top 20 keywords in exosomal drug delivery system research.

**Rank**	**Keywords**	**Counts**	**Rank**	**Keywords**	**Counts**
1	Extracellular vesicles	366	11	Stem cells	66
2	Drug delivery	264	12	Cancer	63
3	In vitro	149	13	siRNA	61
4	Nanoparticles	132	14	Drug delivery systems	59
5	Cells	108	15	Biogenesis	58
6	Delivery	107	16	Micro vesicles	58
7	Mesenchymal stem cells	106	17	Doxorubicin	18
8	Drug delivery system	71	18	Dendritic cells	54
9	Exosome	69	19	Paclitaxel	54
10	Cell derived exosome	66	20	Therapy	52

## LIST OF ABBREVIATIONS

ACS: American Chemical Society
BBB: Blood-Brain Barrier
CNS: Central Nervous System
EVs: Extracellular Vesicles
IF: Impact Factor
MISEV2018: Minimal Information for Extracellular Vesicle Research 2018
NSFC: National Natural Science Foundation of China
RMT: Receptor-Mediated Transcytosis
WOS: Web of Science
